# Medical Opinion and Movement

**Published:** 1907-05-25

**Authors:** 


### Medical Opinion and Movement.

It has been held hitherto that, owing to the bactericidal action of the blood serum, it is not generally possible to cultivate the typhoid bacillus from the blood of a patient, such as is taken in the ordinary way for a Widal reaction. According, however, to the latest experiments, this appears to be not so difficult a matter. In a research carried out in the Hygienic Institute in Kiel, typhoid or paratyphoid bacilli were found in the clotted blood sent for Widal's reaction in eight out of eleven examinations. These examinations were made by Dr. R. Müller and Dr. H. Graf, and similar results have since been obtained by other observers. Dr. Fornet has succeeded in cultivating the organisms fourteen times out of nineteen examinations of these blood clots in capillary tubes. He adopted the method of incubating the clots in test-tubes containing sterilised ox-bile. The suggestion is that the bile dissolves the clot and so liberates the organism. By this means the diagnosis of typhoid may be established in the first few days of the disease, when the Widal reaction is slight or altogether negative. It is just in these early days that such a test is of the greatest assistance to the clinician.

Many efforts are now being made to improve the sanitary conditions in Ireland and to rouse the people to a sense of the importance of questions of public health for their well-being and prosperity. The latest endeavour on these lines is the Women's National Health Association of Ireland, which has been organised by the Countess of Aberdeen. The objects of this Association are (1) to arouse public opinion, and especially that of the women of Ireland, to a sense of responsibility regarding the public health; (2) to spread the knowledge of what may be done in every home and by every householder to guard against disease and to eradicate it when it appears; (3) to promote the upbringing of a healthy and vigorous race. The problems which immediately demand the attention of the Association are those affecting the prevention of consumption, the reduction in infant mortality, the provision of a pure supply of milk, the medical inspection of children in the elementary schools, the teaching of the principles of health to the children in school, and the improvement in school sanitation. Her Excellency the Countess of Aberdeen is President and Honorary Treasurer, and Mrs. Rushton is the Organising Secretary in Dublin. Branches are to be formed in different parts of Ireland. We wish every success to such an excellent movement, and hope it may bear good fruits in the immediate future.

One of the most harassing elements in the life of a general practitioner is the fact that he is always "on duty." Even the Sunday rest is often denied to him, and the cherished hope of repose after a week of incessant work and worry proves delusive, and the new week has to be faced unrefreshed and unrested. On the Continent efforts are being made to ensure more leisure time on Sunday to the medical man. At the recent Congress of Medical Practitioners in Paris the question was debated, and a resolution was passed in favour of charging double fees for Sunday visits. Since then Dr. Ravon, of St.-Etienne, has dealt with the subject, and is of opinion that the public require educating on the question, and that they should be charged at the rate of night visits after midday on Sundays and holidays. There seems no reason why medical men in this country should not also take the matter in hand, and make it known to the public that they also require a day of rest, and expect to be left in peace except in so far as cases of urgent necessity may arise. Most medical men will probably agree with Dr. Ravon's computation that only about 25 per cent. of the visits requested by patients on Sundays are really necessary. The idea that a medical man requires a Sunday rest simply does not occur to the majority of people, and so Sunday afternoon appears to be a convenient time, with both parents at home, to discuss the ailments of any member of the family with the doctor. The visit would probably not appear so desirable if it was known that a double fee would be charged.

Dr. Robert Saundby, Professor of Medicine in the University of Birmingham, has recently discussed in an interesting paper that somewhat rare condition known as splenomegalic polycythæmia. The disease is characterised by a progressive asthenia, accompanied by high blood pressure, general redness or cyanosis of the skin, splenic enlargement, and considerable increase in the number of red corpuscles, with a proportional rise in the hæmoglobin content, the specific gravity, and the viscosity of the blood, but without any increase in the number of white cells. Dr. Saundby gives details of two cases which have come under his observation in the Birmingham Hospital, one of which has already been published by Dr. Russell in 1902. He then proceeds to discuss the etiology. Contrary to most other observers, who have placed the primary lesion in the bone marrow and regard the polycythæmia as the essential factor of the disease, Dr. Saundby is inclined to the view that this increase in red corpuscles is of a compensatory nature and secondary to the stagnation in the capillary circulation resulting from a contraction of the middle-sized arteries and arterioles. In support of this view he points to the increase of red corpuscles which may occur in cases of congenital heart diseases and other conditions of deficient oxygenation of the blood. He puts forward, therefore, the somewhat ingenious theory that in this disease the primary lesion consists in a nervous vasomotor spasm caused by some toxin, possibly influenzal in origin. This continual spasm leads to pronounced thickening of the arterial walls, with engorgement of the capillary and venous circulation and congestion of the internal organs; and this vascular stagnation and general asphyxial condition call forth increased activity of the bone marrow and consequent polycythæmia, which in turn further impedes the capillary circulation.

